# Anticholinergic Burden Scoring in Orthogeriatric Patients: A Two-Cycle Quality Improvement Audit

**DOI:** 10.7759/cureus.117986

**Published:** 2026-10-15

**Authors:** Yashwanth Sooranahalli Nabh, Ayesha Hamid

**Affiliations:** 1 General Medicine, Ashford and St. Peter's Hospitals NHS Foundation Trust, Surrey, GBR; 2 Trauma and Orthopedics, Ashford and St. Peter's Hospitals NHS Foundation Trust, Surrey, GBR

**Keywords:** anticholinergic burden, clinical audit, cognitive impairment, medication review, older adults, orthogeriatrics

## Abstract

Background: Anticholinergic burden is relevant to medication review in older surgical patients, but routine documentation may be incomplete.

Objective: The objective of this study is to compare documented Anticholinergic Cognitive Burden (ACB) assessment before and after a multicomponent quality-improvement intervention.

Methods: This single-centre retrospective quality-improvement audit compared two independent cohorts of surgical patients aged ≥65 years in June 2023 and April 2024. Staff education, posters, and a proforma incorporating ACB and medication-review prompts were introduced between cycles. Process outcomes were compared using Fisher’s exact test. The pooled cross-sectional association between ACB and the composite cognitive-screen outcome was exploratory and unadjusted.

Results: Seventy-two patients were included: 33 at baseline and 39 at re-audit. ACB documentation increased from 0/33 (0.0%) to 15/39 (38.5%), an absolute increase of 38.46 percentage points (95% CI 21.35-54.10; p<0.001). Documented medication review increased from 1/33 (3.0%) to 19/39 (48.7%; p<0.001), and documented medication modification from 1/33 (3.0%) to 10/39 (25.6%; p=0.009). ACB-category distributions did not differ significantly (p=0.910). The composite cognitive outcome was present in 16/25 (64.0%) patients with ACB ≥2 and 7/47 (14.9%) with ACB 0-1, corresponding to an unadjusted prevalence ratio of 4.30 (95% CI 2.04-9.04). This association was limited by the small sample, different preoperative cognitive instruments, and absence of confounder adjustment.

Conclusion: Documented ACB assessment and medication-review processes increased between cycles, but implementation remained incomplete. A reduction in medication burden or improvement in patient-centred outcomes was not established.

## Introduction

Anticholinergic medicines inhibit acetylcholine at muscarinic receptors, and anticholinergic burden describes the cumulative contribution of these medicines within a treatment regimen [1,2]. Polypharmacy makes this relevant to older adults [2,3], whose altered pharmacokinetics and reduced cholinergic reserve may increase susceptibility to adverse effects [1]. Frailty and pre-existing cognitive impairment further complicate assessment [3,4].

Potential adverse effects include peripheral symptoms and central cognitive disturbance [1,5]. Observational evidence links anticholinergic exposure with cognitive impairment, falls, hospitalisation and mortality, although associations vary across medicines, populations and measurement scales [6-9]. Confounding and heterogeneous findings limit causal interpretation, and evidence that reducing burden improves patient-centred outcomes remains uncertain [5,10].

The Anticholinergic Cognitive Burden (ACB) scale provides a practical, widely used approach to identifying cumulative exposure during medication review [11]. Its additive scoring is compatible with a documentation-focused audit, but the scale does not fully incorporate dose, duration or individual susceptibility, and alternative instruments are not interchangeable [1,11,12]. In orthogeriatric care, medication exposure coexists with perioperative precipitants of cognitive disturbance [7,13]. ACB scoring can therefore prompt review of treatment indication, expected benefit and alternatives [14]; it should not mandate automatic deprescribing [12,14].

The local practice gap was the absence of documented ACB scores in the June 2023 baseline cohort, alongside infrequent documented medication review. Previous studies describe anticholinergic exposure in hospital and nursing-home populations and support examining structured medication-review approaches [3,15,16]. However, feasibility and improved recognition should be distinguished from demonstrated clinical benefit [5,17]. Staff education, posters and an ACB-related proforma prompt were introduced before re-audit in April 2024.

The primary improvement aim was to increase the proportion of eligible orthogeriatric surgical patients with a clinician-documented ACB score between June 2023 and April 2024. This process measure was selected because a score calculated retrospectively by the audit team does not establish its recognition during clinical care. Documented medication review and medication modification were separate secondary process outcomes; cognitive-screening completion and ACB distribution were also described. The pooled association between ACB and the composite cognitive outcome was a distinct exploratory analysis. A prospectively agreed numerical compliance target cannot be established from the available project documentation; consequently, the audit evaluates change from baseline rather than attainment of a retrospectively assigned target.

## Materials and methods

Study design and setting

This was a single-centre, retrospective, two-cycle clinical audit and quality-improvement project conducted within the orthogeriatric service at a single NHS trust. The baseline audit examined patients admitted in June 2023, and the re-audit examined patients admitted in April 2024 following the implementation of targeted quality-improvement measures. The project evaluated whether the interventions improved the assessment and documentation of ACB, medication review, and medication modification among older surgical patients. The project is reported in accordance with the principles of the Standards for Quality Improvement Reporting Excellence (SQUIRE 2.0) [18].

Study population

Patients were eligible if they were aged 65 years or older, were admitted under the orthogeriatric service during either audit period, and underwent a surgical procedure. Patients were excluded if they were younger than 65 years, did not undergo surgery, were receiving palliative care at admission, represented a duplicate admission, or had records lacking the minimum information required to determine the principal audit outcomes. All eligible patients identified during the two audit periods were included. The baseline cycle comprised 33 patients, and the re-audit comprised 39 patients, producing a combined sample of 72 patients.

Data collection and variable definitions

Data were retrospectively extracted from available clinical records, medication charts, cognitive assessment documentation, and admission and discharge records using a standardised data-collection form. The extracted characteristics included age, sex, dementia history, admission and discharge dates, length of hospital stay, number and names of regular medicines, cognitive assessments, evidence of cognitive impairment, documented medication review, medication modification, and clinician documentation of an ACB score. Patient identifiers were replaced with study identification numbers, and the audit dataset was stored in a password-protected format. Where possible, extracted records were checked by a second reviewer for accuracy and consistency.

The audit-calculated ACB score was distinguished from clinician documentation of the score. For the audit calculation, every regular medicine was reviewed against the ACB scale, the relevant score for each contributing medicine was assigned, and the medicine-level scores were summed to obtain the patient’s total ACB score. Each medicine was counted only once per patient. Total scores were classified into four mutually exclusive categories: ACB 0, ACB 1, ACB 2, and ACB 3 or greater. An ACB score of 3 or greater was treated as the principal threshold for elevated anticholinergic burden, while a threshold of ACB 2 or greater was also examined as an exploratory grouping. The names and frequencies of individual ACB-contributing medicines were recorded to identify the most frequently encountered agents.

Cognitive screening was considered completed when at least one preoperative Abbreviated Mental Test Score (AMTS), preoperative 4AT, or postoperative 4AT assessment was documented. Cognitive impairment was operationally defined as an AMTS below 7 and/or a 4AT score of 4 or greater. Preoperative AMTS was documented in 28/33 patients (84.8%) in the baseline cycle, whereas preoperative 4AT was documented in 39/39 patients (100.0%) in the re-audit. Postoperative 4AT was documented in all patients in both cycles: 33/33 (100.0%) at baseline and 39/39 (100.0%) at re-audit. A 4AT score of 4 or greater was interpreted as indicating possible delirium or cognitive impairment requiring clinical assessment and was not considered an independent confirmation of delirium.

Quality-improvement interventions

Following completion of the June 2023 baseline audit, the findings were presented to the clinical team. The quality-improvement programme subsequently included staff education, the display of educational posters, and the introduction of a clinical proforma incorporating an ACB-assessment field and medication-review prompt. These measures were intended to increase recognition of anticholinergic burden and encourage clinicians to review potentially contributory medicines. A re-audit was conducted in April 2024 to evaluate changes following these interventions. The sequence is shown schematically in Figure 1; the spacing between activities does not represent the exact elapsed time.

**Figure 1:**
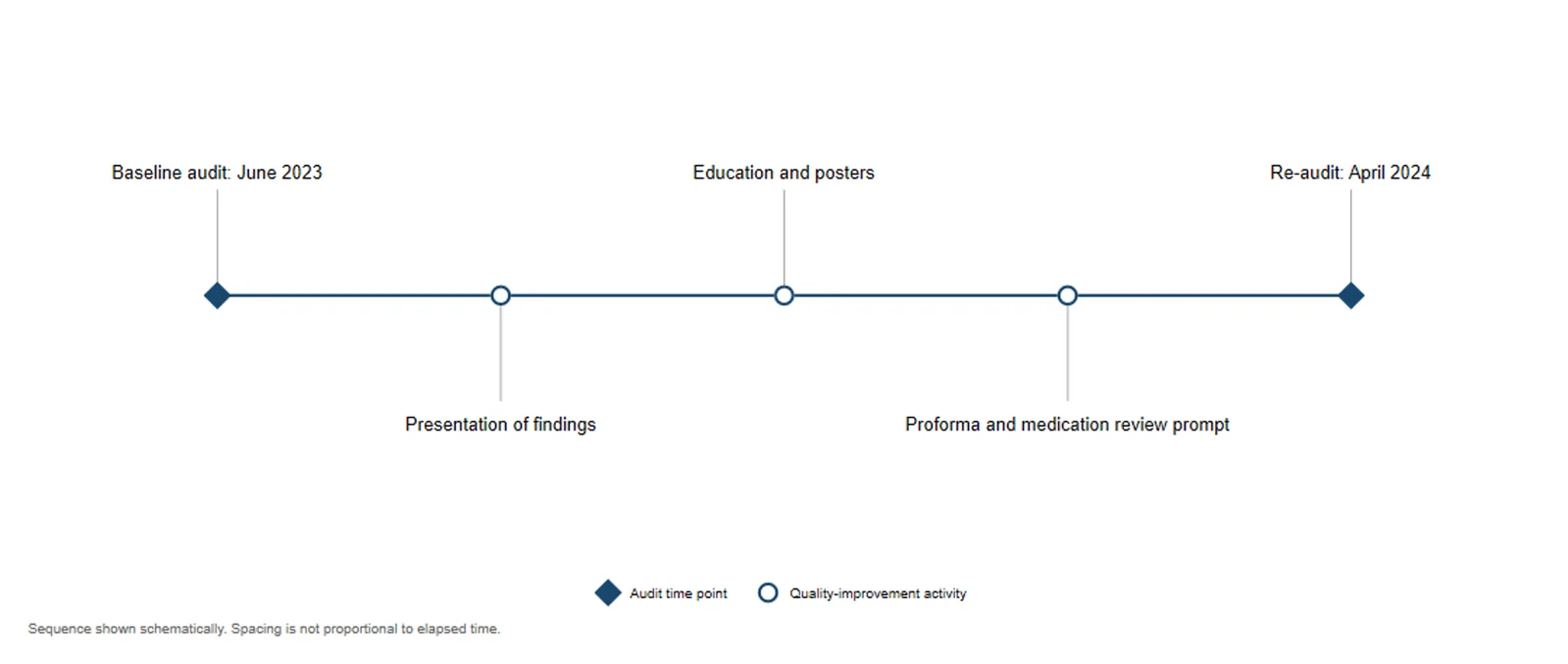
Study and quality-improvement timeline. The baseline audit in June 2023 was followed by presentation of the findings, staff education and posters, and implementation of a proforma and medication-review prompt before the April 2024 re-audit. Diamonds denote audit time points, while circles denote quality-improvement activities. The sequence is schematic and not proportional to elapsed time. This figure presents the intervention sequence rather than quantitative data; summary statistics, statistical tests, and a significance threshold are therefore not applicable.

Outcome measures

The primary process outcome was explicit documentation of an ACB score in the clinical record. Secondary process outcomes were documented medication review, documented medication change, and completion of cognitive screening. A medication review was considered documented when the clinical record showed assessment of the medicine’s indication, dose, potential interactions, anticholinergic burden, or possible lower-burden alternatives. Medication change was defined as the documented discontinuation, dose reduction, or substitution of at least one ACB-contributing medicine. Cycle-specific proportions for all process measures were calculated using the full number of eligible patients in each cycle.

Additional descriptive and exploratory outcomes included patient demographic and clinical characteristics, audit-calculated ACB-score distribution, the most frequently identified ACB-contributing medicines, and the prevalence of cognitive impairment. The exploratory association between ACB and cognitive impairment was assessed using both ACB 2 or greater versus ACB 0-1 and ACB 3 or greater versus ACB below 3.

Statistical analysis

Categorical variables were summarised as frequencies and percentages. Age was presented as mean (standard deviation), while regular medication count, length of stay, and total ACB score were presented as median (interquartile range). Age was compared between the independent audit cohorts using Student’s t-test assuming equal variances. Regular medication count, length of stay, and total ACB score were compared using two-sided Mann-Whitney U tests, with a normal approximation incorporating adjustment for ties and continuity correction. The t statistic and degrees of freedom were reported for age, and U statistics were reported for the June 2023 group. Binary categorical outcomes were compared using two-sided Fisher’s exact tests because of small cell frequencies. The overall ACB-category distribution was compared using the extension of Fisher’s exact test for a 2×4 contingency table. Fisher’s exact tests do not yield a t, χ², or F statistic. Differences in process outcomes were expressed as absolute percentage-point changes, calculated as April 2024 minus June 2023, with 95% confidence intervals calculated using Newcombe’s method based on Wilson score intervals. Error bars for individual proportions represent Wilson 95% confidence intervals.

The relationships between elevated ACB and cognitive impairment were expressed as unadjusted risk ratios (RRs) with 95% confidence intervals (CIs), calculated using the normal approximation on the logarithmic RR scale. Multivariable adjustment was not undertaken because of the limited sample size and the exploratory nature of this analysis. Patients with complete ACB and cognitive-impairment information were included in these analyses; complete data were available for all 72 patients. No missing values were imputed. Statistical tests were two-sided, and a value of p<0.05 was considered statistically significant. Analyses were performed using Stata 19 (StataCorp, College Station, Texas, USA).

## Results

Patient characteristics

A total of 72 patients were included: 33 in the June 2023 baseline audit and 39 in the April 2024 re-audit. The cohorts had similar demographic and clinical characteristics. Mean age was 84.18 years (SD 7.07) at baseline and 84.03 years (SD 7.06) at re-audit. Women represented 25 of 33 patients (75.8%) and 29 of 39 patients (74.4%), respectively, while a history of dementia was documented in six patients (18.2%) and five patients (12.8%).

The median number of regular medicines was eight in both cycles. The median hospital length of stay was 13 days (IQR 9-16) in June 2023 and 12 days (IQR 9-16) in April 2024. Cognitive assessment was completed for every patient in both cycles. Cognitive impairment was identified in 12 of 33 patients (36.4%) at baseline and 11 of 39 patients (28.2%) at re-audit. The median audit-calculated ACB score was 1 (IQR 0-2) in both cycles. No statistically significant between-cycle differences were detected for the characteristics presented in Table 1.

**Table 1:** Demographic and clinical characteristics by audit cycle. Data are presented as patient counts (n), counts with percentages (n (%)), counts relative to the relevant denominator (n/N (%)), mean (SD), or median (IQR), as indicated. Values in parentheses following medians represent the first and third quartiles. Percentage denominators are 33 patients for June 2023 and 39 for April 2024. Age was compared using an independent-samples Student’s t-test assuming equal variances (t=0.093, df=70; p=0.926). Regular medication count, length of stay, and total ACB score were compared using two-sided Mann–Whitney U tests with tie adjustment and continuity correction: U=643.5, p=1.000; U=685.5, p=0.638; and U=645.5, p=0.986, respectively. U statistics refer to the June 2023 group. Categorical comparisons used two-sided Fisher’s exact tests, which do not yield a t, χ², or F statistic. Statistical significance was defined as p<0.05. No statistical comparison was performed for the number of patients. ACB, Anticholinergic Cognitive Burden; SD, standard deviation; IQR, interquartile range; df, degrees of freedom; N/A = Not applicable.

Characteristic	June 2023 (N=33)	April 2024 (N=39)	p-value
Patients, n	33	39	N/A
Age, mean (SD), years	84.18 (7.07)	84.03 (7.06)	0.926
Female sex, n (%)	25 (75.76)	29 (74.36)	1.000
Dementia history, n (%)	6 (18.18)	5 (12.82)	0.744
Regular medicines, median (IQR)	8 (6–10)	8 (6–10)	1.000
Length of stay, median (IQR), days	13 (9–16)	12 (9–16)	0.638
Cognitive assessment completed, n (%)	33 (100.00)	39 (100.00)	1.000
Cognitive impairment, n/N (%)	12/33 (36.36)	11/39 (28.21)	0.613
Total ACB, median (IQR)	1 (0–2)	1 (0–2)	0.986

ACB score distribution and contributing medicines

The distribution of ACB scores was broadly similar between the audit cycles. In June 2023, 11 patients (33.3%) had an ACB score of 0, 10 (30.3%) had a score of 1, 9 (27.3%) had a score of 2, and 3 (9.1%) had a score of 3 or greater. The corresponding numbers in April 2024 were 13 (33.3%), 13 (33.3%), 8 (20.5%), and 5 (12.8%), respectively. There was no evidence of an overall difference in ACB-category distribution between the cycles (Fisher’s exact test, p=0.910).

At least one ACB-contributing medicine was identified in 22/33 patients (66.67%) in June 2023 and 26/39 (66.67%) in April 2024. An ACB score ≥3 was recorded in 3/33 (9.09%) and 5/39 (12.82%) patients, respectively, representing 8/72 (11.11%) patients overall (Table 2).

**Table 2:** Distribution of Anticholinergic Cognitive Burden scores and prevalence of contributing medicines by audit cycle. Data are presented as patient counts (n) and percentages (%) in separate columns. Percentage denominators are 33 patients for June 2023, 39 for April 2024, and 72 for the overall cohort. ACB categories are mutually exclusive; the final row summarises patients receiving at least one contributing medicine and is not an additional ACB category. This table is descriptive; no hypothesis tests are presented. The study-wide threshold for statistical significance was p<0.05. ACB, Anticholinergic Cognitive Burden.

Measure	June 2023 n	June 2023 %	April 2024 n	April 2024 %	Overall n	Overall %
ACB 0	11	33.33	13	33.33	24	33.33
ACB 1	10	30.3	13	33.33	23	31.94
ACB 2	9	27.27	8	20.51	17	23.61
ACB ≥3	3	9.09	5	12.82	8	11.11
At least one ACB-contributing medicine	22	66.67	26	66.67	48	66.67

Codeine was the most frequently identified ACB-contributing medicine in June 2023, whereas lansoprazole was most frequent in April 2024. Overall, codeine and lansoprazole were each recorded in 12 patients (16.67%), followed by citalopram in nine patients (12.50%) (Table 3).

**Table 3:** Most frequently identified ACB-contributing medicines by audit cycle and in the overall cohort. Data are presented as patient counts (n) and percentages (%) in separate columns. Percentage denominators are 33 patients for June 2023, 39 for April 2024, and 72 for the overall cohort. Each medicine was counted no more than once per patient; patients could receive more than one contributing medicine. The table lists the most frequently reported medicines, including ties, rather than all contributing medicines. Citalopram and lansoprazole were equally frequent in June 2023; atenolol, citalopram, famotidine, and furosemide were tied for third place in April 2024; codeine and lansoprazole were equally frequent overall. This table is descriptive; no hypothesis tests are presented. The study-wide threshold for statistical significance was p<0.05. ACB, Anticholinergic Cognitive Burden.

Cohort	Medicine	Patient count, n	Percentage, %
June 2023	Codeine	7	21.21
June 2023	Citalopram	5	15.15
June 2023	Lansoprazole	5	15.15
April 2024	Lansoprazole	7	17.95
April 2024	Codeine	5	12.82
April 2024	Atenolol	4	10.26
April 2024	Citalopram	4	10.26
April 2024	Famotidine	4	10.26
April 2024	Furosemide	4	10.26
Overall	Codeine	12	16.67
Overall	Lansoprazole	12	16.67
Overall	Citalopram	9	12.50

The within-cycle ACB-score distributions are displayed in Figure 2. The visual pattern confirms that the overall distribution remained relatively stable between audit cycles.

**Figure 2:**
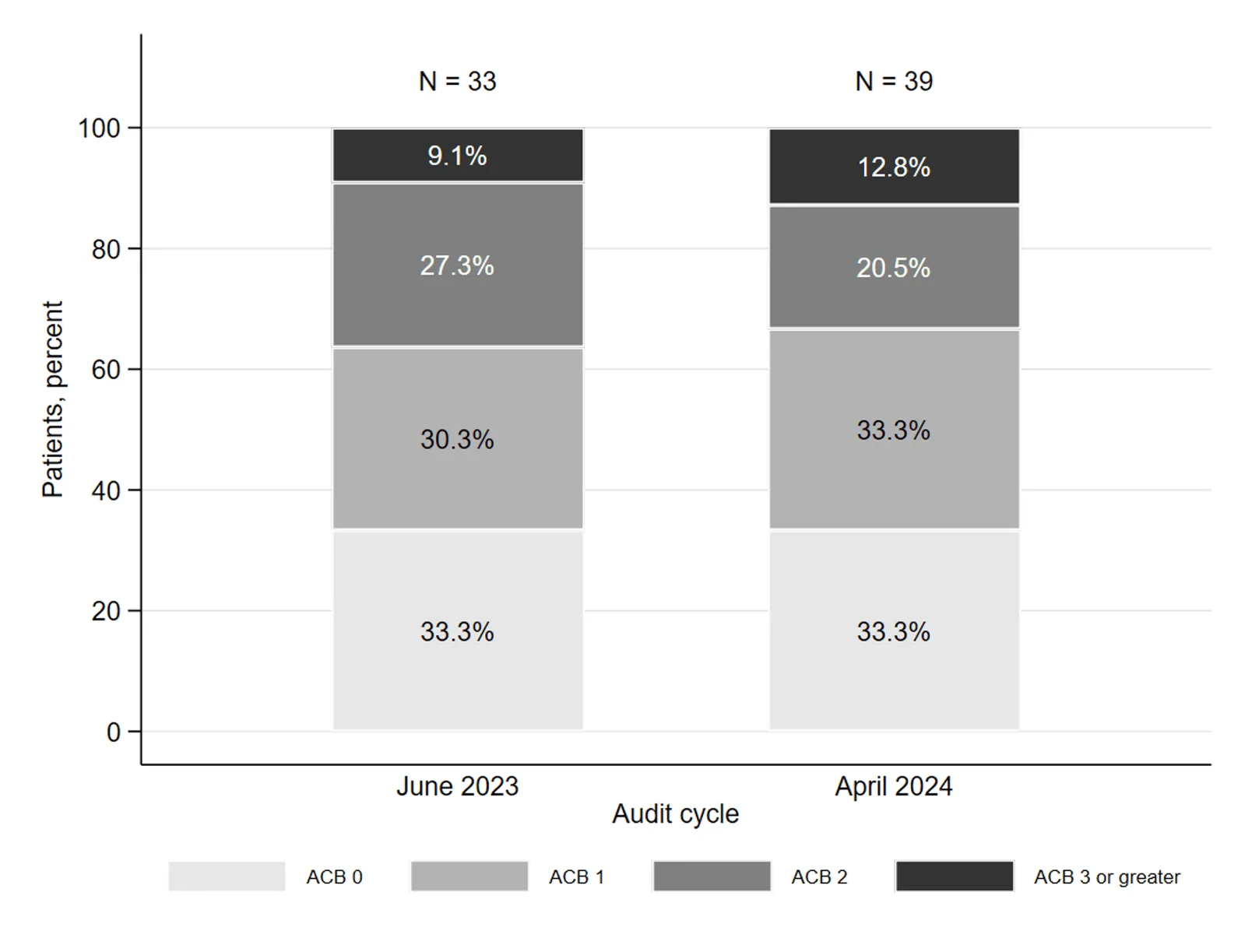
Anticholinergic Cognitive Burden score distribution by audit cycle. The 100% stacked bars show within-cycle patient percentages (%); corresponding counts and percentages are provided here as n/N (%), where n is the category count and N is the total number of patients in the audit cycle. In June 2023 (N=33), the distributions were ACB 0: 11/33 (33.3%); ACB 1: 10/33 (30.3%); ACB 2: 9/33 (27.3%); and ACB ≥3: 3/33 (9.1%). In April 2024 (N=39), the distributions were ACB 0: 13/39 (33.3%); ACB 1: 13/39 (33.3%); ACB 2: 8/39 (20.5%); and ACB ≥3: 5/39 (12.8%). Percentages are rounded and may not sum to exactly 100%. The overall distribution was compared using the extension of Fisher’s exact test for a 2×4 contingency table (p=0.910). This exact test does not yield a t, χ², or F statistic. Statistical significance was defined as p<0.05. ACB, Anticholinergic Cognitive Burden.

Process outcomes following the quality-improvement interventions

Improvements were observed in three of the four process measures following implementation of the quality-improvement interventions. Explicit ACB-score documentation increased from 0 of 33 patients (0.0%) in June 2023 to 15 of 39 patients (38.5%) in April 2024, an absolute improvement of 38.46 percentage points (95% CI 21.35-54.10; p<0.001).

Documented medication review showed the largest improvement, increasing from 1 of 33 patients (3.0%) to 19 of 39 patients (48.7%), corresponding to an absolute increase of 45.69 percentage points (95% CI 26.41-60.98; p<0.001). Documented medication changes increased from 1 of 33 patients (3.0%) to 10 of 39 patients (25.6%), an absolute increase of 22.61 percentage points (95% CI 6.07-38.25; p=0.009). Cognitive screening remained fully documented in both cycles, occurring in 33/33 patients (100.0%) at baseline and 39/39 (100.0%) at re-audit, with no observed change (Fisher’s exact p=1.000) (Table 4).

**Table 4:** Comparison of process outcomes between the baseline audit and re-audit. Data are presented as n/N (%), where n is the number meeting the process measure and N is the total number of patients in the audit cycle. Absolute changes are presented in percentage points with 95% confidence intervals, calculated using Newcombe’s method based on Wilson score intervals. Changes were calculated as the April 2024 percentage minus the June 2023 percentage; positive values indicate improvement. All patients within each audit cycle were included in the denominator. Between-cycle comparisons used two-sided Fisher’s exact tests, which do not yield a t, χ², or F statistic. Statistical significance was defined as p<0.05. ACB, Anticholinergic Cognitive Burden; CI, confidence interval.

Process measure	June 2023, n/N (%)	April 2024, n/N (%)	Change, percentage points (95% CI)	p-value
ACB score documented	0/33 (0.00)	15/39 (38.46)	38.46 (21.35 to 54.10)	<0.001
Medication review documented	1/33 (3.03)	19/39 (48.72)	45.69 (26.41 to 60.98)	<0.001
Medication changed	1/33 (3.03)	10/39 (25.64)	22.61 (6.07 to 38.25)	0.009
Cognitive screening documented	33/33 (100.00)	39/39 (100.00)	0.00 (−8.97 to 10.43)	1.000

Figure 3 illustrates the improvements in ACB-score documentation, medication-review documentation and medication modification. The error bars show uncertainty around each proportion; interval widths reflect both the sample size and the observed proportion.

**Figure 3:**
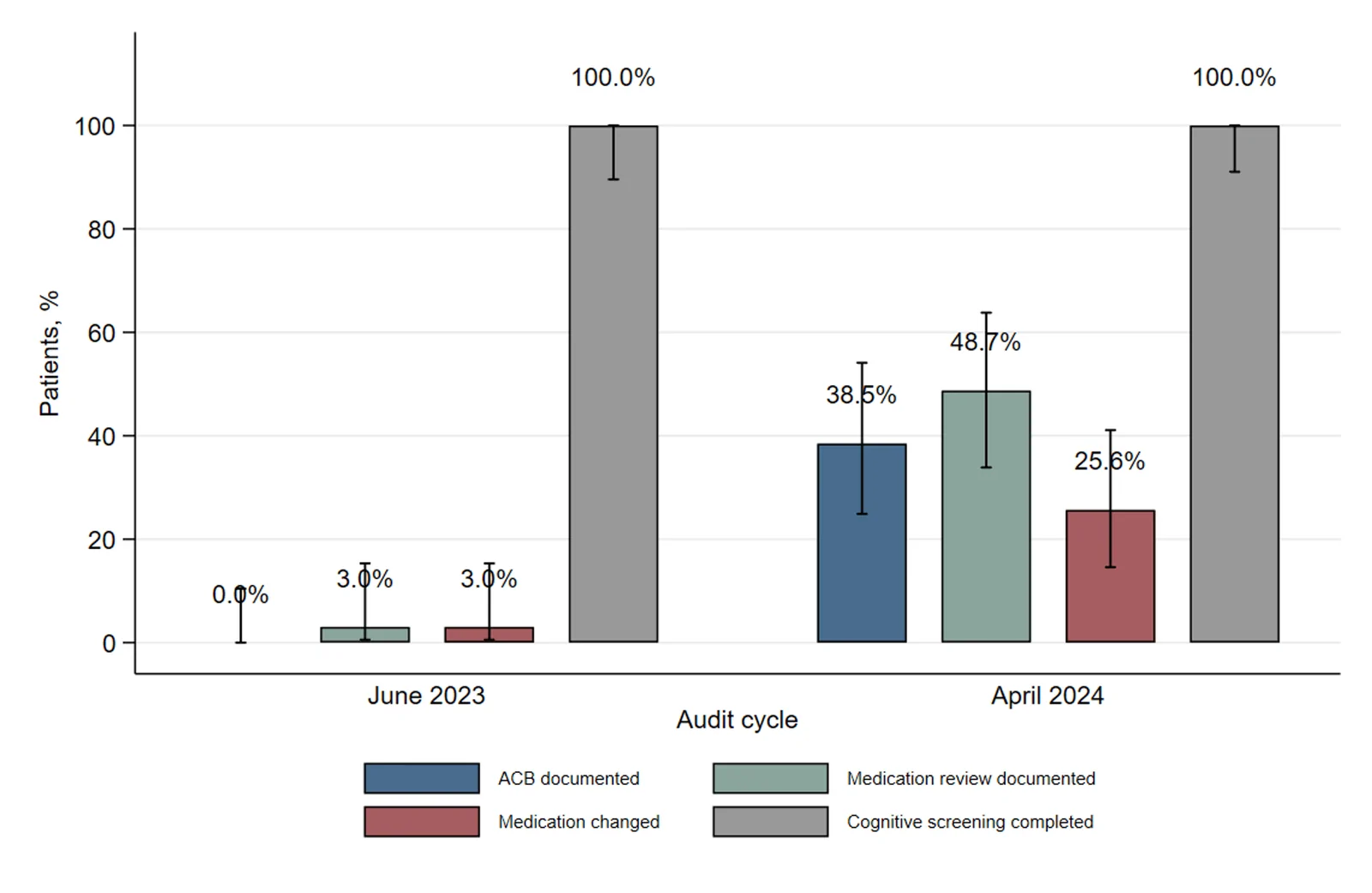
Comparison of process outcomes between audit cycles. Grouped bars show patient percentages (%), with error bars representing Wilson 95% confidence intervals for individual proportions. Corresponding counts and percentages are reported as n/N (%), where n is the number meeting the process measure and N is the audit-cycle total. For June 2023 (N=33) and April 2024 (N=39), respectively, ACB-score documentation occurred in 0/33 (0.0%) and 15/39 (38.5%); medication-review documentation in 1/33 (3.0%) and 19/39 (48.7%); medication change in 1/33 (3.0%) and 10/39 (25.6%); and completed cognitive screening in 33/33 (100.0%) and 39/39 (100.0%). Between-cycle comparisons used two-sided Fisher’s exact tests: ACB documentation, p<0.001; medication-review documentation, p<0.001; medication change, p=0.009; and cognitive screening, p=1.000. Fisher’s exact test does not yield a t, χ², or F statistic. Statistical significance was defined as p<0.05. ACB, Anticholinergic Cognitive Burden.

Association between ACB and cognitive impairment

Cognitive impairment was identified in 4 of 24 patients (16.7%) with an ACB score of 0 and 3 of 23 patients (13.0%) with a score of 1. Its prevalence was substantially higher among patients with greater anticholinergic burden, affecting 10 of 17 patients (58.8%) with an ACB score of 2 and 6 of 8 patients (75.0%) with a score of 3 or greater.

The category-specific pattern is displayed in Figure 4. Confidence intervals were wider in the higher-score categories because of their smaller sample sizes. When ACB scores were dichotomised at 2, cognitive impairment was identified in 7 of 47 patients (14.9%) with an ACB score of 0-1 and 16 of 25 patients (64.0%) with an ACB score of 2 or greater. An ACB score of 2 or greater was associated with a more than fourfold higher unadjusted risk of cognitive impairment compared with an ACB score of 0-1 (RR 4.30, 95% CI 2.04-9.04; Fisher’s exact p<0.001).

**Figure 4:**
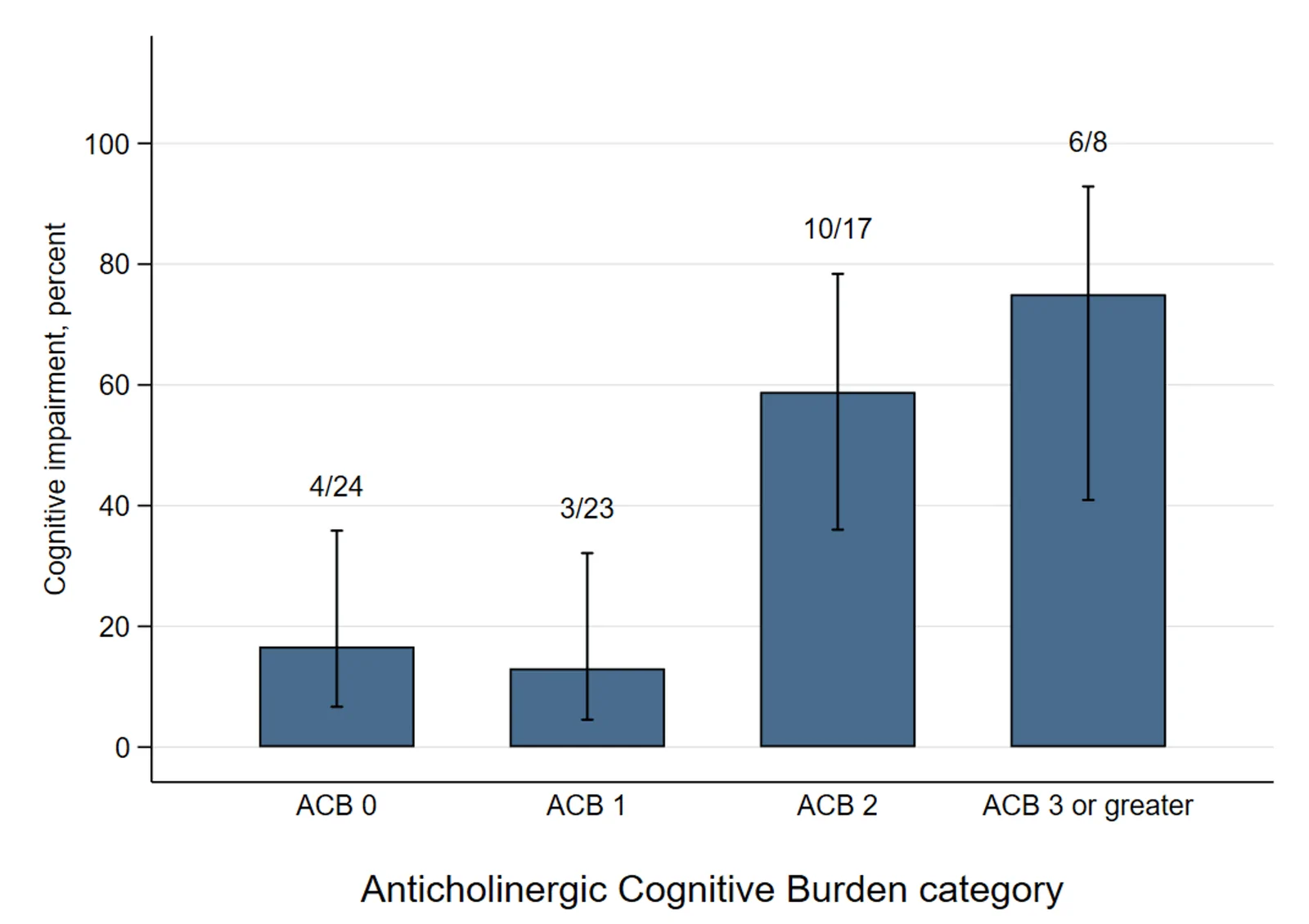
Cognitive impairment according to the Anticholinergic Cognitive Burden category. Bars show the percentage (%) of patients with cognitive impairment within each ACB category in the pooled cohort (N=72). Labels above the bars indicate the number with cognitive impairment divided by the category total (n/N). Corresponding counts and percentages were ACB 0: 4/24 (16.7%); ACB 1: 3/23 (13.0%); ACB 2: 10/17 (58.8%); and ACB ≥3: 6/8 (75.0%). Error bars represent Wilson 95% confidence intervals for individual proportions. For the separately analysed dichotomised comparisons, cognitive impairment occurred in 16/25 (64.0%) patients with ACB ≥2 versus 7/47 (14.9%) with ACB 0–1 (unadjusted RR 4.30, 95% CI 2.04–9.04; p<0.001), and in 6/8 (75.0%) patients with ACB ≥3 versus 17/64 (26.6%) with ACB <3 (unadjusted RR 2.82, 95% CI 1.60–5.00; p=0.011). These p-values relate to the two dichotomised comparisons, not an overall test across the four plotted categories. Both comparisons used two-sided Fisher’s exact tests, which do not yield a t, χ², or F statistic. Statistical significance was defined as p<0.05. ACB, Anticholinergic Cognitive Burden; CI, confidence interval; RR, risk ratio.

Using the prespecified elevated-burden threshold, cognitive impairment was present in 17 of 64 patients (26.6%) with an ACB score below 3 and 6 of 8 patients (75.0%) with an ACB score of 3 or greater. This corresponded to an approximately threefold higher unadjusted risk among patients with an ACB score of 3 or greater (RR 2.82, 95% CI 1.60-5.00; Fisher’s exact p=0.011) (Table 5).

**Table 5:** Association between ACB and cognitive impairment. Data are presented as patient counts (n), counts with within-category percentages (n (%)), and unadjusted risk ratios with 95% confidence intervals calculated on the logarithmic RR scale. Each higher-ACB category is compared with its corresponding reference category. The two dichotomised comparisons use overlapping groups from the same pooled cohort and are not independent analyses. p-values were calculated using two-sided Fisher’s exact tests, which do not yield a t, χ², or F statistic. Statistical significance was defined as p<0.05. Complete ACB and cognitive-impairment data were available for all 72 patients. No separate p-value is assigned to a reference row. ACB, Anticholinergic Cognitive Burden; CI, confidence interval.

ACB category	Total, n	Cognitive impairment, n (%)	Risk ratio (95% CI)	p-value
ACB 0–1	47	7 (14.89)	Reference	Not applicable
ACB ≥2	25	16 (64.00)	4.30 (2.04–9.04)	<0.001
ACB <3	64	17 (26.56)	Reference	Not applicable
ACB ≥3	8	6 (75.00)	2.82 (1.60–5.00)	0.011

## Discussion

The principal finding of this single-centre audit was an increase in documented ACB assessment between two independent cohorts, from 0.0% at baseline to 38.5% at re-audit. Documented medication review and modification also increased. These findings describe changes in recorded processes following the intervention; they do not establish that the intervention caused the changes or that prescribing decisions or patient outcomes improved. ACB documentation remained absent in most re-audit records, and fewer than half documented a medication review. Routine implementation was therefore incomplete.

Interpretation of the process findings

Prior reports examine hospitalisation in cognitively vulnerable patients [19], postoperative delirium in surgical populations [20], automated inpatient alerts [21], associations across different burden scales [22], and mortality [23]. These diverse outcomes provide context but are not substitutes for assessing the present documentation intervention. Education, posters and a proforma prompt offer plausible ways to improve recognition and recording. Similar initiatives have reported improvements following education and routine scoring in an acute geriatric ward [24] and integration into a collaborative frailty clinic [25]. Their populations, implementation methods and outcomes differ from this audit. Because the current components were introduced together, their individual contributions cannot be separated.

There was no concurrent control group. Secular changes, staff turnover, evolving prescribing practice and awareness of the planned re-audit, including possible Hawthorne effects, could have contributed. Attendance, professional-group participation and direct proforma uptake were not quantified in the reported evaluation. In particular, the proportion with a documented ACB score is an outcome measure and cannot be treated as a validated measure of exposure to every intervention component.

The absence of a statistically significant difference in ACB-category distribution neither demonstrates equivalence between cycles nor excludes individual medication changes. Independent cohorts and the absence of paired pre-review and post-review scores prevent attribution of burden changes to review. A dose reduction may leave an additive ACB score unchanged. Conversely, increased documentation alone is insufficient evidence of safer care. These distinctions are consistent with uncertainty about clinical benefits in reviews of burden-reduction interventions [5,16].

Scale selection and clinical context

ACB offers a practical framework for recognising cumulative medication exposure, supported by its established use in older populations [11]. This supports its suitability for a local process audit, but does not establish superiority over the Anticholinergic Risk Scale or Anticholinergic Burden Score. Differences in medicine inclusion and assigned potency can change exposure estimates and associations [11,12,22]. The audit did not compare scales, and the exact historical scale version, access date and complete medicine-level scoring list cannot be verified from the reported methods. Contemporary sources cannot establish which version was actually used during the audit.

Exposure estimates and contributing medicines vary across hospital, psychiatric, memory-clinic and surgical populations [2,3,19,20]. The medicines most frequently reported in this audit should therefore be interpreted within the selected scoring system and clinical setting. Similarities with other inpatient reports [21] do not independently validate individual medicine scores. ACB-guided review should consider therapeutic indication, benefit, alternatives and withdrawal risk [12,14]. Continuing a medicine after an appropriate assessment may be reasonable; a higher rate of medication modification is not, by itself, proof of better prescribing.

Interpretation of the exploratory cognitive analysis

The pooled analysis describes the prevalence of a composite cognitive-screen outcome across ACB categories. Although the unchanged statistical reporting uses the term risk ratio, the estimates compare observed proportions in cross-sectional data and are interpreted here as unadjusted prevalence ratios, not incident risks. They do not establish whether medication exposure preceded cognitive impairment or whether reducing ACB would improve cognition. The comparisons at ACB ≥2 and ACB ≥3 use overlapping participants from the same 72-patient cohort and are correlated exploratory contrasts, not independent replications. No additional clinical action threshold is established by these comparisons.

The wider literature provides biological and clinical context but cannot remove confounding from this audit. Associations have been reported in observational and surgical studies [6,20,22], while differences between scales and attenuation after adjustment illustrate sensitivity to measurement and case mix [7,19]. Longitudinal associations with adverse outcomes [8,9,23] likewise do not demonstrate that changing a score reverses those outcomes. Reviews in cognitively vulnerable populations emphasise uncertainty [5,10]. Accordingly, the cognitive finding remains secondary to the documentation objective.

Strengths and limitations

Strengths include completion of two audit cycles, clear separation of audit-calculated scores from clinician documentation, explicit denominators, and confidence intervals and exact tests suited to small categorical samples. The single-centre setting, short sampling periods and small independent cohorts nevertheless restrict precision and generalisability. Similar measured characteristics do not exclude differences in dementia severity, frailty, polypharmacy, acute illness, surgical factors or prescribing indication.

Retrospective documentation can miss assessments and discussions that occurred but were not recorded. Conversely, a record meeting the audit definition of medication review does not verify that the review occurred as described or was comprehensive, clinically appropriate or implemented. Review quality and adherence were not independently validated. The reported data do not establish whether medication modifications were concentrated in high-burden patients or distributed across ACB categories; aggregate improvement should not be interpreted as evidence of targeted deprescribing.

The scoring concerned documented regular medicines at the time of record review, rather than paired admission and post-review exposure. It cannot establish a uniform preoperative exposure window or the temporal ordering of medication use and cognitive findings. Coverage of over-the-counter medicines and supplements was not established. Restriction to regular medicines also does not capture cumulative as-needed administration. Omitting such exposure may underestimate total burden; the magnitude and direction of its effect on the observed association cannot be quantified. Additive ACB scoring further omits important information about dose, duration, adherence, active metabolites and individual susceptibility [1,12].

Preoperative assessment differed between cycles: AMTS was recorded in 28/33 baseline patients, whereas preoperative 4AT was recorded in all 39 re-audit patients. The 4AT is a rapid clinical screening tool for delirium and cognitive impairment; a score of at least 4 suggests possible delirium and requires clinical assessment rather than establishing a diagnosis. AMTS-defined impairment and a positive 4AT screen are related but non-equivalent constructs. Combining them may introduce outcome misclassification. Common postoperative 4AT assessment in all patients does not remove this heterogeneity and explains the ceiling in the broad screening-completion measure. The available reporting does not establish whether the change in preoperative instrument was planned or reflected changing clinical practice.

Dementia history was described but was not accounted for through adjustment, exclusion or a reported stratified analysis. Confounding by dementia, frailty, polypharmacy, acute illness and prescribing indication, as well as reverse causation, remains substantial. Cycle-, instrument- and dementia-specific sensitivity analyses were not reported, and they cannot be reconstructed reliably from the published marginal counts. Only eight patients had ACB ≥3. Pooling increases the descriptive sample size but does not make the cohorts or screening approaches equivalent.

Further reporting limitations concern the absence of a complete screening and exclusion log, a quantified independently checked proportion, inter-reviewer agreement, detailed intervention delivery measures and a verifiable prospective numerical target. The available reporting also does not establish whether formal sample-size or precision planning or a dated analysis plan preceded analysis. These limitations constrain reproducibility and cannot be resolved by assigning plausible values retrospectively.

Human participant data and governance

This audit analysed retrospective patient-level clinical records. Patient identifiers were replaced with study identification numbers, and the audit dataset was stored in a password-protected format. These safeguards do not themselves establish an ethics exemption or consent waiver. Institutional audit registration, the formal ethics or service-evaluation determination, data-governance authorisation, and consent or waiver status cannot be verified from the available project documentation.

Sustainability and future evaluation

The available evaluation ends with the April 2024 cycle. Sustained improvement, any subsequent audit activity and the reasons for an interval without reported follow-up cannot be established. Electronic prompts, recurring education and greater pharmacy involvement are potential future strategies requiring evaluation, rather than benefits demonstrated here. A further cycle could specify a prospective target, record intervention uptake, use a consistent cognitive instrument, and collect paired medication scores and clinically confirmed outcomes. Balancing measures should assess withdrawal effects, symptom control and medicine reinstatement. Without these data, this audit cannot establish reductions in delirium, falls, length of stay, readmission, functional decline or mortality.

## Conclusions

Documented ACB assessment, medication review and medication modification increased between the June 2023 and April 2024 audit cycles, but routine implementation remained incomplete. The uncontrolled comparison of independent cohorts does not establish that the intervention caused these changes. A reduction in anticholinergic burden or improvement in patient-centred outcomes was not demonstrated; the pooled cognitive association remains exploratory and unadjusted.
